# Psychometric evaluation and validation of Urdu Social Rank Scale for women with infertility in Pakistan

**DOI:** 10.3389/fpsyt.2023.1150941

**Published:** 2023-08-30

**Authors:** Anila Sadaf Mubashir, Syeda Shahida Batool, S. M. Yasir Arafat

**Affiliations:** ^1^Applied Psychology National University of Modern Languages NUML, Islamabad, Pakistan; ^2^Department of Psychology, Government College University, Lahore, Pakistan; ^3^Department of Psychiatry, Enam Medical College and Hospital, Dhaka, Bangladesh

**Keywords:** social rank, infertility, social comparison, submissive behavior, psychometric evaluation

## Abstract

**Background:**

Infertility negatively affects nearly all aspects of women’s life and is a source of demotion in the rank/status of women that they have achieved after marriage. This social rank/status demotion due to infertility may result in depression and several other psychopathologies. No extant instrument is available to measure the phenomenon of social rank in women with infertility in Pakistan.

**Objective:**

The aim of the current study was to evaluate the psychometric properties and validate the Social Rank Scale for women experiencing infertility in Pakistan.

**Methodology:**

This study was conducted in four phases. The data were collected from women with primary infertility who visited hospitals all over Pakistan from 2016 to 2018. Social Rank Scale for Women with Infertility (SRS-WI) comprising of two scales, the Social Comparison Scale for Women with Infertility (SCS-WI) and the Submissive Behavior Scale for Women with Infertility (SBS-WI), was developed.

**Results:**

The factor structure of 37 items of SCS-WI and of 21 items of SBS-WI was determined through exploratory factor analysis (EFA) on a sample of 215 women with primary infertility with an age range of 20–45 years (*M_age_* = 31.03; SD = 6.18). Principal component analysis with varimax rotation method yielded a three-factor solution for SCS-WI, and 32 items were retained for SCS-WI that accounted for 62.38% variance. For SBS-WI, a uni-factor solution was obtained, and 20 items were retained for SBS-WI, which collectively accounted for 42.01% variance. The factor structure for both scales was confirmed via confirmatory factor analysis among a sample of 210 participants with good model fit indices.

**Conclusion:**

The study provides acceptable psychometric properties of the SRS-WI in Pakistan. Testing of psychometric properties in different groups of samples would justify the generalized use of the instrument.

## Introduction

Social rank is directly associated with reproductive ability amongst married people in Pakistani society (1). Psychological repercussions of infertility include stigmatization, seclusion, depression, anxiety, and guilt (2, 3). Pakistani women face persistent pressure to bear a son for the sake of the continuation of lineage (4). Failure to do so imposes labels such as “barren and cursed” on them (5). Women perceive an insurmountable portion of psychological distress compared to men (6).

Cross-cultural differences in the perception of infertility are rather prominent, as in Eastern societies deficient social support results in high levels of psychopathological outcomes (7). A feeling of inferiority and consequential loss of social rank lead to depressive symptoms owing to hopelessness and worthlessness (8). Pakistani women with primary infertility are subjected to systematic and progressive devaluation, as child-bearing is a prerequisite for strengthening their foothold in the in-laws’ house (9). For this reason, it is imperative to assess this phenomenon. Social rank is measured by social comparison and submissive behavior variables. Social comparison is comprised of perceptions of personal superiority or inferiority (10, 11), and submissive behavior consists of inhibition in situations of challenge or conflict (12–14).

Keeping in view the significance of the phenomenon of social rank and its variables in relation to the detrimental effects caused by a medical condition like primary infertility, there is a strong need to assess this phenomenon in women. In the West, scales such as the Scale of Social Comparison Submissive (15), Submissive Behavior Scale (16) and INCOM by Gibons and Bunk (17) are available with their local norms, but no such scale is available in Pakistan to assess the variable of social rank, i.e., social comparison and submissive behavior, especially with respect to infertility. Although two self-report scales of social rank, i.e., Social Comparison Scale (SCS) and Submissive Behavior Scales (SBS) were developed in the English language for the student population language in the West and are available, there are certain reservations to using them directly in the Pakistani population, as firstly, they are developed in Western culture according to their norms, and the Pakistani culture has different norms and values. Secondly, both scales are in English, and in all the provinces of Pakistan, the most commonly used and comprehendible language is Urdu. Therefore, we need self-report scales that are easier to understand by most Pakistani women who can read or understand the Urdu language. Thirdly, the available scale of social rank variables, i.e., SCS (15) and SBS (16), are generic tools and cannot give us a true picture of the perception of social rank variables with special reference to primary infertility in women of Pakistan. So, it was felt that a self-report infertility-specific scale of social rank in the context of Pakistani culture needed to be developed.

## Materials and methods

The study was conducted in four phases.

### Phase I: generation of an item pool

An initial item pool in the Urdu language was generated using both inductive (semi-structured interviews) and deductive approaches. The item pool was then presented to a committee comprised of five judges (three assistant professors and two lecturers from the Department of Psychology) to determine content validity. After consensus, 37 items were retained for the SCS for Women with Infertility, and 21 items were reserved for the SBS for Women with Infertility based on a strict selection criterion: construct reliability, the perception, the precision of statement, comprehensibility, clarity, and redundancy. The arrangement of the items was shuffled by arranging the items from general to more defined and specified content.

The format of the response by the participants for the 37 items in the SCS-WI (18) was categorized to be a Likert type 5-point scale (0 = strongly disagree, 1 = disagree, 2 = indecisive, 3 = strongly agree, 4 = strongly agree). The response format of the 21 items SBS-WI (19) was also decided to be 5-point Likert type scale (0 = Never, 1 = Rarely, 2 = Sometimes, 3 = Mostly, 4 = Always).

### Phase II: pilot study

The pilot study was conducted for the psychometric scrutiny of the items (removing the ambiguous, repeatedly occurring and redundant items) and to test the initial scale to ensure lucidity and comprehensibility of the items’ statements.

The study was carried out by recruiting 30 women with primary infertility from Rawalpindi city by using purposive sampling strategy. The age range of the participants was 20 to 45 years (*M_age_* = 31.37, *SD* = 5.18).

The participants were thus informed about the purpose of the current investigation and the procedure of affirmative consent. Respondents were asked to answer the questions of the survey on the spot, and their queries were catered to accordingly.

The integral purpose of this preliminary study was to grasp the comprehensibility and clarity of the content of the scale and consequently finalize the items for the exploratory factor analysis for phase III of this study. In order to determine the normality of the data, the Shapiro–Wilk test was incorporated, and values W (30) = 0.94, *p* > 0.05 of SCS-WI and values W (30) = 0.96, *p* > 0.05 indicated that there was normal distribution of data within the group. Some of the items were modified after getting feedback from the participants. For example, item number 1 was re-phrased to “I feel physically inferior as equated to child bearing mothers” from “I feel inferior as compared to child bearing mothers.” Item 9 was rephrased from “I understand that I am given less importance in the family in comparison to child bearing mothers” to “I understand that as compared to child bearing mothers, I do not have that much influence in the family.” Item 11 was rephrased from “I understand that I am not as likeable as child bearing mothers are.” Item 28 was rephrased to “I understand that I am not as likeable in the family as compared to child bearing mothers” from “Due to infertility, I feel more helpless and cry.” In Item 30, the word people were replaced by “family.” However, there was no deletion done for any single one of the items. For SBS-WI, item 1 was rephrased to “I feel inferior due to infertility” from simply “I feel inferior.” It was the judges’ mutual consent to add “due to infertility” in all item statements.

Ultimately, 37 items for Social Comparison Scale for Women with Infertility (SCS-WI) with reverse scoring for items 6 and 7 and maximum score of 148 were finalized. Further, 21 items for the Submissive Behavior Scale for Women with Infertility (SBS-WI) with a maximum score of 84 were finalized.

### Phase III: dimensionality and internal consistency

This phase was conducted for finding the factorial validity of SCS-WI and SBS-WI. Both the scales were analyzed in order to finalize the statements that could be retained after assuring the appropriate structure. Cronbach’s alpha, item-total scale correlation, and item subscale correlation were computed to conclude the reliability and internal consistency of the overall scale.

The sample of the current study was recruited through a purposive sampling, and 230 women with primary infertility were selected from all major cities of different provinces of Pakistan. Participants of the study fall in the age range of 20–45 years (*M_age_ = 31.03; SD = 6.18*). The mean duration of infertility period was approximately 8 to 9 years (*M = 8.88; SD = 6.52*). The average number of treatments sought by the women in this study was six (*M = 5.76; SD = 6.35*). Participants of the study had formal educational levels from primary to post-masters level and belonged to a variety of socioeconomic classes. On the whole, the participants were quite cooperative; however, eight participants dropped out of the study for personal reasons while six respondents were unable to return the survey, so their data was excluded from the study. The final sample consisted of 215 women with infertility. Overall response rate of the participants was 93.4%.

Instruments used in study were demographic sheet, 37-item Social Comparison Scale for Women with Infertility (SCS-WI), with a response format of a 5-point Likert scale and 21 item Submissive Behavior Scale for Women with Infertility (SBS-WI). Participants were encouraged to fill out all the questionnaires during the current investigation, and their queries related to survey forms were acknowledged and answered then and there for their satisfaction.

Before officially carrying out Exploratory Factor Analysis, the assumptions and rules of Factor Analysis were empirically tested for checking the data to be factor analyzed and were found satisfactory for both SCS-WI and SBS-WI. The sample adequacy for SCS-WI (37-item) was tested with Kayser-Myer sample of adequacy measure (KMO, Kaiser, 1960). The KMO value was 0.95, and it was excellent *χ*^2^(496) = 5606.98, *p* < 0.001. Moreover, significant Bartlett’s test also assured the factorability.

For SBS-WI, the sampling adequacy was 0.93 for The Kaiser-Meyer-Olkin measure which was depicted as excellent for structure detection, while the Bartlett’s Test of Sphericity was highly significant χ^2^(210) =2390.33, *p* < 0.001 with a prominent indication that factor analysis had been proved appropriate for the current data. Hence, the study proceeded forward. The normality of the SCS-WI and SBS-WI items was checked by computing the skewness and kurtosis, as factor analysis is robust to the assumptions of normality. It was found that all the items were in the range of acceptable levels for SCS-WI and SBS-WI. It was assured that before running the factor analysis, no potential missing values were present in the data; for furthering our study, this assumption was found to be satisfactory. The computed item correlation matrix of SCS-WI and SBS- WI indicated that all the items were suitable for structure detection. All the communality values of SCS-WI and SBS-WI were within the acceptable range. There were no outliers found in the data of SCS-WI and SBS-WI revealed by the box plot.

Results of factor analysis in Table 1 yielded a three-factor solution through principal component analysis and a fixed factor solution followed by orthogonal rotation (varimax method) with Eigen values greater than 1.0. Out of 37 items, 32 items showed factor loadings on three factors. On the basis of scree plot, factor loadings greater than 0.40 in each subscale and theoretical relevance, three were well defined, interpretable, clear accurate factors were retained. These factors labeled as Social Distress (F1: factor 1), Emotional Burden (F2: factor 2) and Personal Incapacity (F3: factor 3). The Eigen value for factor 1 is 16.35, explaining 51.11% of variance; factor 2 is 2.46, explaining 8.24% of variance, and factor 3 is 1.41, explaining 4.41% of variance, and totally they accounted for 63.76% of the total variance. Out of 37 items, 5 items (items no. 6, 7, 8, 31, and 36) were deleted that had factor loading less than 0.40 with Eigen value <1.0 (item 6 “I am affable”; item 7 “I feel more attractive as compared to child bearing mothers.” Item 8 “I feel more helpless as compared to child bearing mothers” has same content as item 28 “I cry over my helplessness more than child bearing mothers” which was rephrased and retained. Item 31 “Due to infertility my husband does not give importance to me” because the content was nearly the same as item 22 “My husband does not cooperate with me due to infertility/childlessness.” Item 36 “I avoid social gatherings as compared to other women”). No item required reverse coding, as negatively phrased items were reported as confusing by the women in pilot study. High score on SCS-WI indicates higher negative and unfavorable social comparison.

**Table 1:** Factor loadings, mean and standard deviation (*N* = 215) of social comparison scale for women with infertility SCS-WI.

Old item no./New item no.	Factors			*M*	*SD*
	1	2	3		
SCS-WI 21/ SCS-WI 18	**0.87**	0.27	0.12	2.20	1.56
SCS-WI 20/ SCS-WI 17	**0.87**	0.21	0.20	2.07	1.56
SCS-WI 25/ SCS-WI 22	**0.85**	0.19	0.19	2.03	1.63
SCS-WI 19/ SCS-WI 16	**0.79**	0.27	0.26	2.20	1.53
SCS-WI 22 /SCS-WI 19	**0.77**	0.26	0.25	2.23	1.55
SCS-WI 26/SCS-WI 23	**0.75**	0.23	0.25	2.38	1.54
SCS-WI 35/SCS-WI 30	**0.71**	0.32	0.20	2.47	1.51
SCS-WI 18/SCS-WI 15	**0.67**	0.22	0.45	2.49	1.45
SCS-WI 24/SCS-WI 21	**0.67**	0.45	0.17	2.56	1.56
SCS-WI 23/SCS-WI 20	**0.64**	0.46	0.23	2.07	1.56
SCS-WI 30/SCS-WI 32	**0.59**	0.20	0.34	2.05	1.50
SCS-WI 9/ SC S-WI 6	**0.57**	−0.13	0.28	2.20	1.53
SCS-WI 11/SCS-WI 8	**0.53**	0.30	0.51	2.50	1.41
SCS-WI 27/SCS-WI 24	**0.43**	−0.31	−0.14	1.65	1.71
SCS-WI 15/SCS-WI 12	**0.42**	−0.08	−0.34	1.59	1.61
SCS-WI 33/SCS-WI 28	0.15	**0.78**	0.13	3.33	1.08
SCS-WI 32/SCS-WI 26	0.23	**0.76**	0.11	3.27	1.07
SCS-WI 28/SCS-WI 27	0.32	**0.65**	0.36	3.01	1.30
SCS-WI 2/SCS-WI 2	0.07	**0.65**	0.42	2.46	1.45
SCS-WI 5/SCS-WI 5	0.33	**0.61**	0.17	1.59	1.61
SCS-WI 3/SCS-WI 3	0.26	**0.60**	0.45	2.30	1.50
SCS-WI 34/SCS-WI 29	0.28	**0.55**	0.52	2.91	1.29
SCS-WI 37/SCS-WI 31	0.33	**0.53**	0.41	2.99	1.24
SCS-WI 12/SCS-WI 9	0.33	0.17	**0.72**	2.46	1.45
SCS-WI 10/SCS-WI 7	0.33	0.19	**0.72**	2.31	1.51
SCS-WI 13/SCS-WI 10	0.42	0.17	**0.71**	2.30	1.50
SCS-WI 14/SCS-WI 11	0.35	0.30	**0.71**	2.54	1.49
SCS-WI 1/SCS-WI 1	0.01	0.44	**0.62**	2.50	1.41
SCS-WI 4/SCS-WI 4	0.26	0.48	**0.59**	2.54	1.49
SCS-WI 16/SCS-WI 13	0.17	0.47	**0.54**	3.28	1.02
SCS-WI 17/SCS-WI 14	0.34	0.45	**0.50**	3.05	1.20
SCS-WI 29/SCS-WI 25	0.41	0.44	**0.45**	2.03	1.63
Eigen values	16.35	2.64	1.41		
% of variance	51.11	8.24	4.41		

Communality > 0.40 (Field, 2005).

*Factor 1 (Social Distress)*. Items 6, 8, 12, 15, 16, 17, 18, 19, 20, 21, 22, 23, 24, 30, and 32 had independent loading on factor 3, and it represents social distress (social factors that have an impact on women with infertility such as social comparison, diminished social status, diminished respect and importance in family, social stigmatization, negative and critical attitude of people, reduced liking in family especially in-laws, rejection by family, blaming by family, reduction in love and attention by husband, left out by husband, cold attitude of husband, uncooperative husband towards treatment, threats of being expelled from husband’s home, threats of second marriage by husband and in-laws, distant relations with husband, gap in communication with husband, in-laws thinking of women with infertility as useless and take her for granted in all household chores and other tasks, social isolation, avoiding social gathering, etc.). It explained 51.11% of variance. *Factor 2 (Emotional Burden)*. Items 2, 3, 5, 26, 27, 28, 29, and 31 had independent loading on factor 2, and Emotional Burden subscale of emotional factors is related to diminished social status of women with infertility, such as feelings of sadness, sorrow, worthlessness, loneliness, helplessness, hopelessness, negative feelings, depression, anger, anxiety, aggression, frustration, irritability, distress, grief, loss of happiness, reduced interest in daily activities, envy, jealousy, insecurity shame, humiliation, etc. It explained 8.24% of variance. *Factor 3 (Personal Incapacity)*. Items 1, 4, 7, 9, 10, 11, 13, 14, 25 had independent loading on factor 1. The items indicate various forms of social comparison of women with primary infertility, such as feelings of inferiority, self-blaming, fatigue, lack of resourcefulness, low self-esteem, low confidence, reduced eye contact, feelings of physical and mental illness and weakness, meaningless life, feeling incomplete, feelings of being left out, negative future apprehensions, reduced attraction, decreased sexual attraction, negligence of personal care and hygiene, etc. It explained 4.41% of variance. The factorial validity of the scale was demonstrated on empirical and theoretical grounds. The final scale came up with 32 items and three well delineated factors, i.e., Social Distress (15 items), Emotional Burden (8 items), and Personal Incapacity (9 items) (Table 1).

*Item-total correlation* of SCS-WI was run to see the internal consistency of the scale. Results indicated that each item of Social Comparison Scale for Women with Infertility correlated (r ranging from 0.44 to 0.78) with the sum of total items. Moreover, mean inter-item correlation was 0.50. Thus, all items are considered valid and reliable indicators of Social Comparison scale for Women with Infertility. Cronbach’s alpha for SCS-WI with infertility (*α* ranging from 0.89 to 0.94), which is good along with the potential range and actual range of the SCS-WI. Furthermore, if we compare the mean of the sample with the potential range of the score on the relevant scale, it appears that on SCS-WI, the mean score is 81.85 and the maximum potential range is 0–128, which indicates that average score of the sample lies on the higher direction showing higher social comparison in women with primary infertility. As far as factor 1, i.e., Social Distress, is concerned, the mean score is 30.10 and the maximum potential range is 0–60, indicating that the score of the sample lies on the average side, whereas a mean score of 21.06 on factor 2, i.e., Emotional Burden, with potential range of 0–36, indicates that the sample mean lies on higher side on this factor. Lastly, the sample mean on factor 3, i.e., Personal Incapacity, is 24.76 with potential range of 0–32, indicating sample mean on higher side in this factor as well. The reliability of this scale (Cronbach’s alpha) was *α* = 0.92 (Table 2).

**Table 2:** Final factors, percentage of variance and alpha coefficients of social comparison scale for women with infertility (*N* = 215).

Factor Label	Final (with new item no.) items retained	Final items	Variance	Alpha coefficients
F1 Social Distress	6, 8, 12, 15, 16, 17, 18, 19, 20, 21, 22, 23, 24, 30, 32	15	51.11%	0.89
F2 Emotional Burden	2, 3, 5, 26, 27, 28, 29, 31	8	8.24%	0.90
F3 Personal Incapacity	1, 4,7, 9, 10, 11, 13, 14, 25	9	4.41%	0.91
SCS-WI	F1, F2, F3	32	63.76%	0.94

Reliability Analysis of SCS-WI.

The 21 items of Submissive Behavior Scale for Women with Infertility (SBS-WI) were factor analyzed. The single factor solution was obtained explaining 42.01% of variance, retaining all the items on the basis of scree plot, and whose factor loading was greater than 0.40 with eigen value >1.0. Only one item, i.e., item number 2 (“I raise voice for my rights”), was discarded, whose factor loading was less than 0.4 with eigen value <1.0. The item-total correlation analysis was performed on 20 items of SBS-WI. The proportion of correlation of each item with the total score of the scale was determined. Results in Table 3 present that each SBS-WI item correlated positively (r ranging from 0.41 to 0.84) and significantly (*p* < 0.01) with the sum of total items; moreover, mean inter item correlation was 0.64. A final 20 items were retained (*M =* 51.00; *SD =* 18.52; response range = 0–4; and skewness = −0.57). Further, if we the compare mean value of the total score of SBS-WI, i.e., 51, with the potential range of 0–80, it appears that it is in higher direction indicating more submissive behavior in women with primary infertility. The item means of almost all the items are also on slightly higher side when compared to potential range, indicating slightly higher submissive behavior in women with primary infertility. Results in Table 3 shows that all items of SBS-WI can be considered valid and reliable indicators of Submissive Behavior Scale in Women with Infertility.

**Table 3:** Item-total correlations for subscales of 32-item social comparison scale for women with infertility (*N* = 215).

Subscales items	*r*	Subscale items	*r*	subscale items	*r*
Social distress		23	0.74	31	0.69
6	0.44	24	0.50	Personal incapacities	
8	0.77	33	0.66	1	0.56
12	0.48	30	0.72	4	0.70
15	0.79	Emotional burden		7	0.69
17	0.79	2	0.57	9	0.68
17	0.78	3	0.70	10	0.73
18	0.77	5	0.60	11	0.75
19	0.78	27	0.73	13	0.62
20	0.76	26	0.56	14	0.71
21	0.76	38	0.55	25	
22	0.74	29	0.74		

All correlations were *p* < 0.01.

#### Inter item correlation of SBS-WI

The item-total correlation analysis was performed on 20 items of SBS-WI. The proportion of correlation of each item with the total score of the scale was determined (Table 4). Results in Table 4 that each SBS-WI item correlated positively (r ranging from 0.41 to 0.84) and significantly (*p* < 0.01) with the sum of total items; moreover, mean inter item correlation was 0.64. A final 20 items were retained (*M =* 51.00; *SD =* 18.52; response range = 0–4; and skewness = −0.57). Further, if we the compare mean value of the total score of SBS-WI, i.e., 51, with the potential range of 0–80, it appears that it is in higher direction indicating more submissive behavior in women with primary infertility. The item means of almost all the items are also on slightly higher side when compared to potential range, indicating slightly higher submissive behavior in women with primary infertility. Results of Table 4 shows that all items of SBS-WI can be considered valid and reliable indicators of Submissive Behavior Scale in Women with Infertility. The reliability of this scale (Cronbach’s alpha) was α = 0.92 (Table 5).

**Table 4:** Factor loadings, item total correlations, mean and standard deviation (*N* = 215).

Old-new item	Factors loading	Item-total correlation (*r*)	Mean	SD	Min	Max
SBS-WI 1–1	**0.48**	0.44	2.24	1.53	0	4
SBS-WI3-2	**0.67**	0.63	2.47	1.43	0	4
SBS-WI4-3	**0.54**	0.50	2.50	1.37	0	4
SBS-WI5-4	**0.44**	0.39	2.71	1.30	0	4
SBS-WI6-5	**0.57**	0.52	2.71	1.26	0	4
SBS-WI7-6	**0.78**	0.75	2.52	1.42	0	4
SBS-WI8-7	**0.56**	0.52	2.39	1.46	0	4
SBS-WI9-8	**0.56**	0.52	2.73	1.33	0	4
SBS-WI10-9	**0.54**	0.50	2.47	1.54	0	4
SBS-WI11-10	**0.64**	0.61	2.33	1.53	0	4
SBS-WI12-11	**0.41**	0.38	2.87	1.31	0	4
SBS-WI13-12	**0.53**	0.50	2.34	1.56	0	4
SBS-WI14-13	**0.59**	0.54	2.21	1.57	0	4
SBS-WI15-14	**0.75**	0.70	2.99	1.18	0	4
SBS-WI16-15	**0.80**	0.74	2.71	1.32	0	4
SBS-WI17-16	**0.83**	0.78	2.62	1.44	0	4
SBS-WI18-17	**0.84**	0.79	2.54	1.49	0	4
SBS-WI19-18	**0.79**	0.73	2.46	1.53	0	4
SBS-WI20-19	**0.78**	0.73	2.66	1.37	0	4
SBS-WI 21–20	**0.77**	0.71	2.54	1.45	0	4
SBS Total	–	–	51.0	18.5	0	80
Eigen value	8.78					
% of variance	42.04					

Bold face Factor loading > 0.40 (Field, 2005).

**Table 5:** Overall Mean, Standard Deviation, Cronbach’s Alpha, Maximum Score, Minimum Score (*N* = 210).

Scale	K	Final Items retained	M(SD)	α	Min.	Max
SCS-WI	27	1-27	55.17(23.48)	.95	19	103
Social distress	11	5, 10, 13, 14, 15, 16, 17, 18, 19,25, 27	21.28(10.23)	.90	3	44
Emotional burden	7	2, 3, 21, 22, 23, 24, 26	14.37(6.35)	.85	1	28
Personal Incapacities	9	1, 4,6, 7, 8, 9, 11, 12, 20	17.43(7.99)	.87	2	33
SBS-WI	17	1-17	28.38(15.33)	.95	0	57
SRS-WI	44	-------------------	81.48(36.70)	.84	19	153

Note. k = no. of items. α = Cronbach’s alpha

### Phase IV: confirmatory factor analysis

To confirm the factor structure and dimensionality of the instruments, the 32 items of Social Comparison Scale for Women with Infertility and 20 items of Submissive Behavior Scale for Women with Infertility were scrutinized through AMOS-21.0. Instruments used were the demographic sheet from the Social Comparison Scale for Women with Infertility (SCS-WI, 32 item). The scale was proven to have good internal reliability with Cronbach’s alphas of 0.79 to 0.95. Submissive Behavior Scale for Women with Infertility (SBS-WI), 20 item with a Cronbach’s alpha of 0.82. Maximum score is 80. Higher scores are indicative of greater feelings of subordination. The study respondents were communicated and informed about the purpose of the current study. Written and affirmative informed consent was taken from all the study participants. It took them approximately 10 to 15 min to complete the questionnaire for the Social Comparison Scale for Women with Infertility and 5 to 10 min to complete the questionnaire for the Submissive Behavior Scale for Women with Infertility. Out of 225 recruited participants, 10 left the study incomplete, and five did not return the questionnaire responses. The final sample consisted of 210 participants.

The current study incorporated numerous indices, standards and criteria to present the best model fit including CFI, TLI, RMSEA, IFI, and GFI, as they have been frequently testified and reported in the recent research (20). The results after confirmatory factor analysis of the Social Comparison Scale for Women with Infertility shows that in the final model, the IFI = 0.91, CFI =0.91 together with χ^2^ (317, *n* = 210) =790.71, *p <* 0.01, and TLI = 0.90, RMSEA = 0.08. In terms of the overall indices, it is evident that this model was acceptable. In initial model, the values of IFI, TLI, CFI, and RMSEA were not in the acceptable range, and the model was revised through modification indices and also by deleting some of items with correlation less than 0.3. Details of items deleted are: Item 5, “I remain quiet most of the time as compared to child bearing mothers,” item 8, “I feel that I am not liked by the family as they do other child bearing mothers,” item 15, “I feel that family members reject me due to childlessness,” item 18, “My husband love for me has decreased due to childlessness,” and item 20, “I feel physically less attractive due to childlessness.” A revised model was obtained, which still required further improvement, as the values of IFI = 0.87, TLI = 0.86, CFI = 0.87, RMSEA = 0.10 were still not in acceptable range. In the final model, covariance as suggested by modification indices were entered in the error terms to further improve the model. Lastly, the final obtained model had all the values in acceptable range (Table 6).

**Table 6:** Standardized CFA solution of social comparison scale for women with infertility, *N* = 210.

Model	IFI	TLI	CFI	RMSEA	*p*	*df*	χ^2^	χ^2^/*df*
Initial Model	0.84	0.83	0.84	0.10	0.000	461	1356.33	2.94
Revised Model	0.87	0.86	0.87	0.10	0.01	321	991.62	3.08
Final Model	0.91	0.90	0.91	0.08	0.01	317	790.71	2.49

IFI, incremental fit index; CFI, comparative fit index; TLI, Tucker Lewis Index Model. Final Model items reduced from 32 to 27 (item nos. 5, 8 15, 18, and 20 deleted).

The results after confirmatory factor analysis of the Submissive Behavior Scale for Women with Infertility (17 items) shows that in the final model, the GFI = 0.90, CFI =0.94 together with χ^2^ (107, *n* = 210) =205.15, *p <* 0.01, and TLI = 0.92, RMSEA = 0.07. In terms of the overall indices, it is evident that this model is acceptable. The initial model obtained has GFI, CLI, TLI, and RMSEA values that do not fall in the acceptable range of model fit indices, so using modification indices the model was improved further and a revised model was obtained that still had values of GFI = 0.86, TLI = 0.87, CFI = 0.89, RMSEA = 0.10 that were not in the acceptable range, so items no. 13, 14, and 18, with correlation less than 0.3, were deleted, and the final obtained model had all the values in the acceptable range. Details of items deleted are item 13, “I avoid eye contact with people due to infertility,” item 14, “I remain quiet when people talk unlikeable things about me,” and item 18, “Due to infertility without any hesitation, family makes me do their work without taking my consent.” In the final model, some covariance was added in error terms to improve the model as recommended by modification indices (Tables 6–8).

**Table 7:** Standardized CFA solution of submissive behavior scale for women with infertility (*N* = 210).

Model	GFI	CFI	TLI	RMSEA	*df*	*p*	χ^2^	χ2/*df*
Initial Model	0.80	0.79	0.76	0.10	170	0.000	528.81	3.11
Revised Model	0.86	0.89	0.87	0.10	119	0.000	398.33	3.34
Final Model	0.90	0.94	0.92	0.07	107	0.01	205.15	1.91

GFI, goodness of fit index; CFI, comparative fit index; TLI, Tucker Lewis Index Model. Final Model items reduced from 20 to 17 (item nos. 13, 14, and 18 deleted).

**Table 8:** Standardized CFA solution of social rank scale (*N* = 210).

Model	IFI	TLI	CFI	*df*	RMSEA	*p*	χ^2^	χ^2^_/_ *df*
Initial Model	0.84	0.83	0.84	898	0.08	<0.000	2039.52	2.27
Final Model	0.90	0.90	0.90	881	0.06	<0.01	1576.13	1.78

IFI, incremental fit index; CFI, comparative fit index; TLI, Tucker Lewis Index.

**Figure 1:**
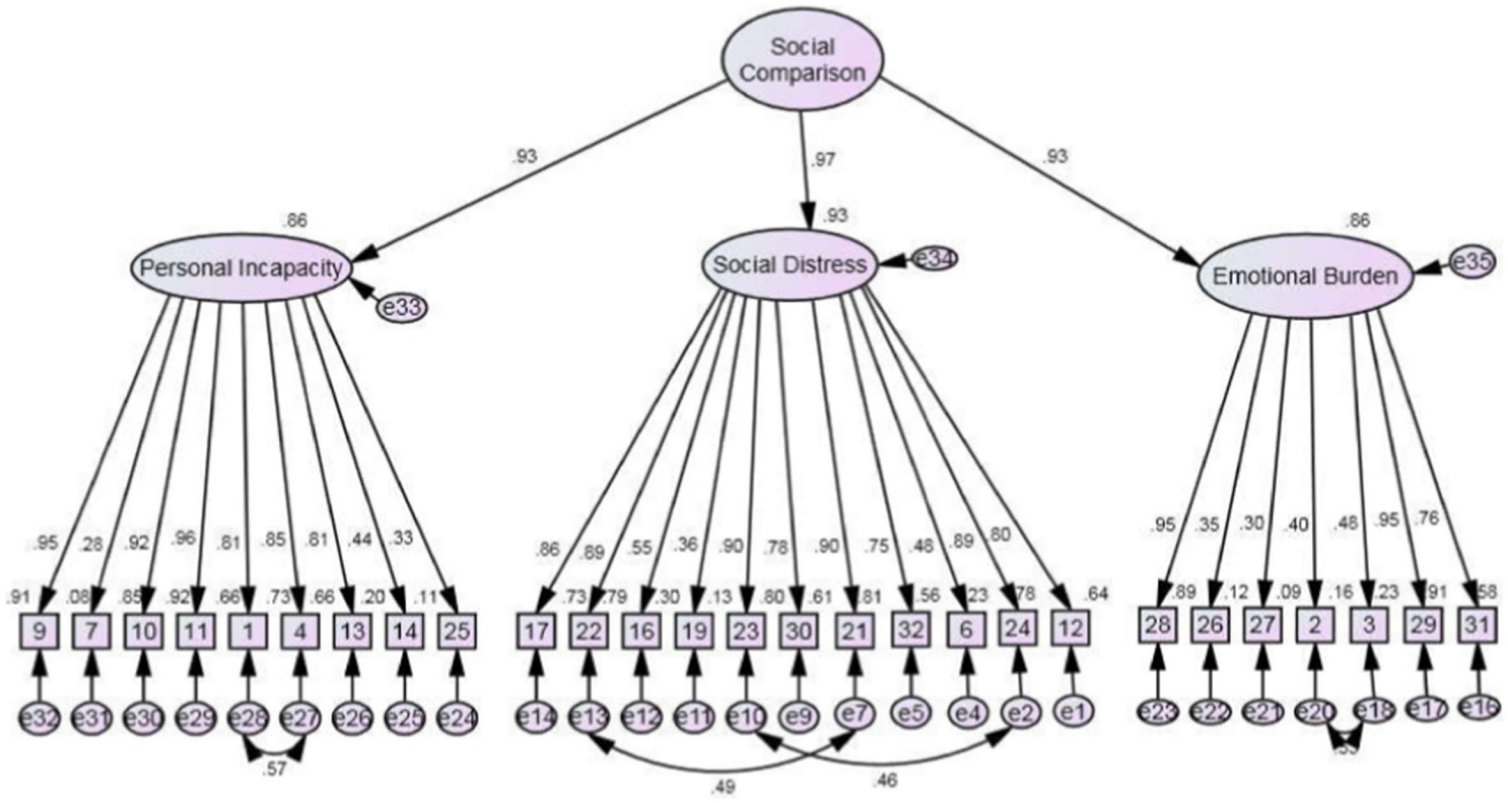
Final model SCS-WI 27-item scale (with modification indices).

**Figure 2:**
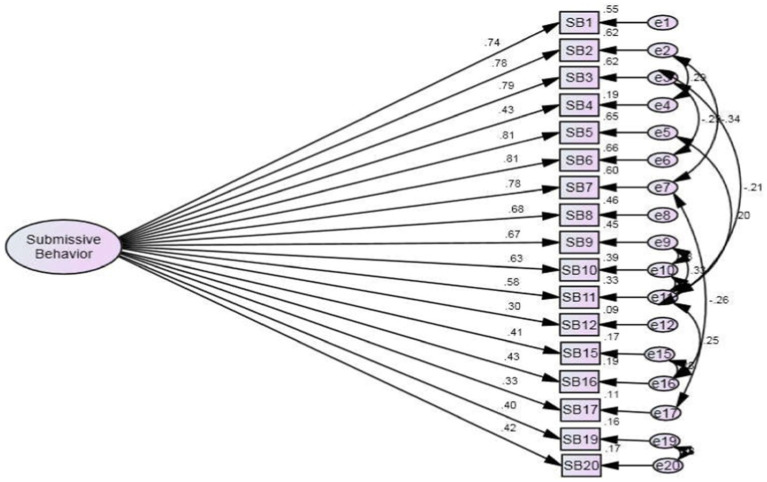
Final model SBS-WI complete standardized solution of submissive behavior scale for Women with infertility (*N* = 210).

**Figure 3:**
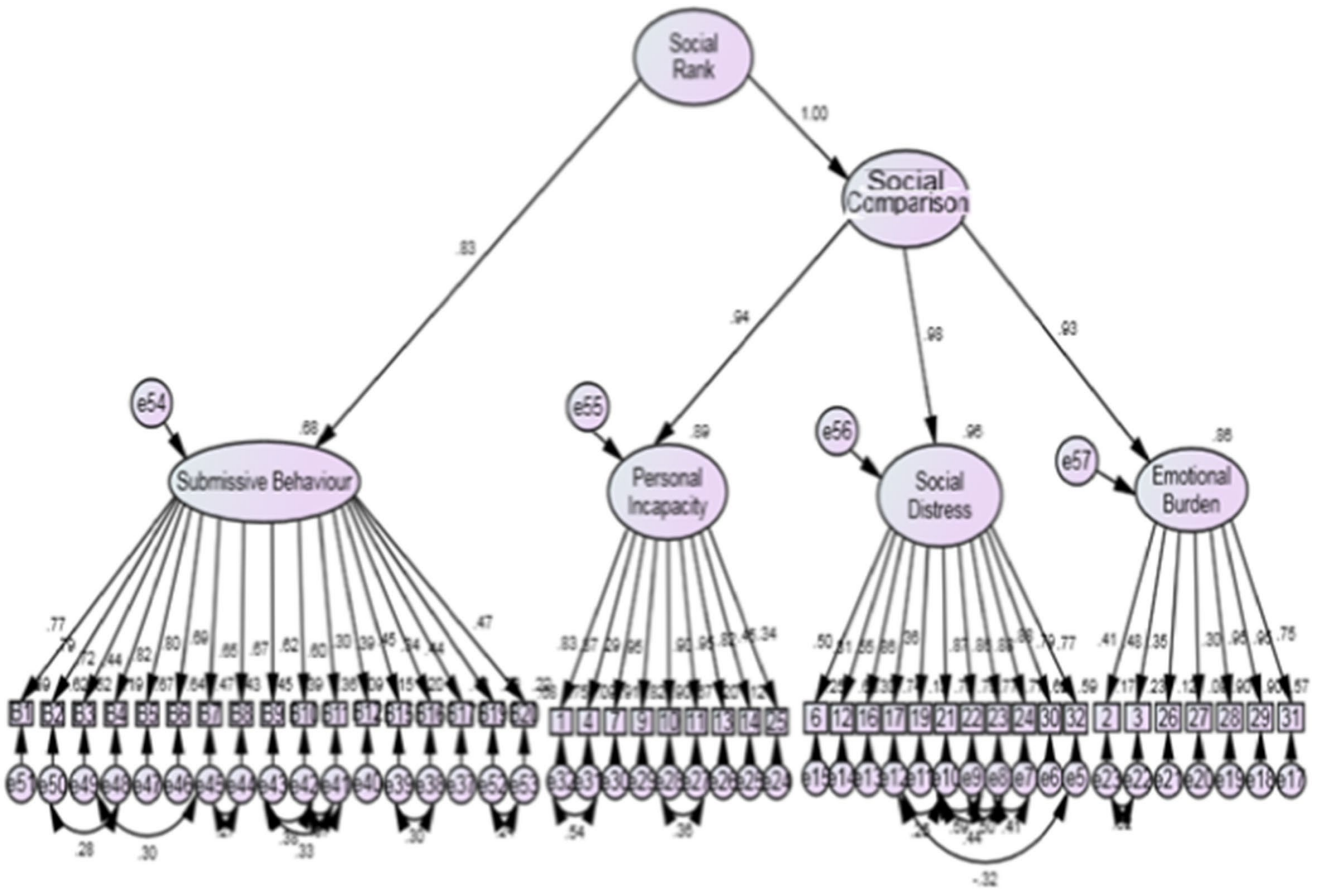
Final model of social rank scale for Women with infertility.

Figures 1–3 indicate that the model supports that Social Rank Scale can be used collectively by including social comparison and submissive behavior independently.

Table 5 indicates the mean, standard deviation, Cronbach’s alpha, and minimum and maximum scores. It is found that overall alpha for Social Comparison for Women with Infertility SCS-WI α = 0.95 (α range from 0.87 to 0.90 for subscales) and Submissive Behavior Scale for Women with Infertility SBS-WI α = 0.95. Overall the alpha reliability of Social Rank Scale is 0.84, which is also good.

#### Convergent and discriminant validity of scales

Convergent validity of SCS-WI was determined by associating it with similarly constructed scales, such as the Social Comparison Scale (15), while convergent validity of SBS-WI was established by correlating it with the Submissive Behavior Scale (16). Taking the research study one step further, the discriminant validity of SCS-WI was determined by correlating it with a theoretically opposing scale, i.e., the Satisfaction with Life Scale (21, 22). Previous studies have shown a negative correlation between social comparison and satisfaction with life, or that low levels of social comparison lead to higher satisfaction with life (23), and discriminant validity of SBS-WI was determined by correlating it with a theoretically opposing scale, i.e., the Self-Assertiveness Scale (24) (previous researchers have shown a negative correlation between submissive behavior and aggressive behavior) (12–14). A purposive sampling technique was employed to recruit a sample of 30 women respondents experiencing primary infertility from different hospitals and clinics in Lahore, Rawalpindi, and Multan city. Instruments used were demographic sheet, the Social Comparison Scale for Women with Infertility (SCS-WI), 27 items, the Submissive Behavior Scale for Women with Infertility, 20 items, and 11 items translated into Urdu from the Social Comparison Scale to assess the internal sense of one’s social ranking by determining how one thinks about where one stands in relation to others. The scale has shown good reliability, with Cronbach’s alpha of 0.87 and 16-item Submissive Behavior Scale. Higher scores indicate greater feelings of subordination. The scale showed good reliability, with a Cronbach’s alpha of 0.78. Furthermore, Urdu translated Satisfaction with Life Scale (5 item) with Cronbach’s alpha for the scale ranges from 0.87 and Self-Assertiveness Scale (28 item). It is used to assess self-assertiveness in adults, through a culture-specific measuring device. It consisted of 28 items with a response option on a 5-point Likert-type scale. A higher score on the scale shows more assertiveness while a low score exhibits less self-assertiveness.

Results of convergent validity studies showed that SCS-WI positively correlated with a similar scale SCS (*r* = 0.37, *p = 0.05), whereas SCS-WI negatively correlated with SWLS (*r = −0.70*, **p < 0.01). The test–retest reliability of SCS-WI after a gap of 1 month on a sample of 30 women with primary infertility was found to be α = 0.93. Pearson product moment correlation was also carried out to find the relationship between Submissive Behavior Scale for Women with Infertility (SBS-WI) and Submissive Behavior Scale (SBS), and SBS-WI and Self Assertiveness Scale (SAS). Results suggested that SBS-WI positively correlated with SBS (*r* = 0.43, *p = 0.05), whereas it negatively correlated with SAS (r = −0.57, **p < 0.01). The test re-test reliability of SBS- WI after an interval of 1 month on a sample of 30 women with primary infertility was found to be *α* = 0.94.

## Discussion

The present study was carried out to develop an indigenous scale of social rank for women with infertility in Pakistan with its two subscales, i.e., the Social Comparison Scale and Submissive Behavior Sale for Women with Infertility, by exploring and confirming the factor structure of the scales and establishing the discriminant validity of the scales, and later in the study keeping in mind the significance of assessing social rank variables in our culture specifically, for women with primary infertility. The need for self-reporting scales in the Urdu language was essential for comprehension. The scales developed in these studies are valuable contributions to assess the phenomenon of social rank through social comparison and submissive behavior in women with infertility.

The scales developed in these studies are valuable contributions to assessing the phenomenon of social rank through social comparison and submissive behavior in women with infertility. The scale development process of the Social Comparison Scale for Women with Infertility (SCS-WI) and the Submissive Behavior Scale for Women with Infertility (SBS-WI) was highly intensive, time consuming, and scientific, and comprised of a mixed method research design. The items for both scales were produced using both inductive and deductive approaches, and the factor structure of the scale was determined through exploratory factor analysis.

The final SCS-WI comprised of 27 items with three well-defined subscales. The first subscale of SCS-WI, i.e., Social Distress, measured the social experiences of women with primary infertility. Participants with high scores on this factor compared themselves unfavorably to childbearing mothers and greatly socially suffered. The items on the scale are based on areas causing social distress. They are related to the status of women with primary infertility in the family, expulsion from home, emotional harassment at home by family members, stigmatization, communal isolation, refusal, fear of rejection, privacy, reduction in love by husband, the uncooperative attitude of husband, and disruption and dissonance in the marital connection due to social and family burden on husband for the need of a second marriage (25). These items are similar to the problems of women with infertility in the Indian subcontinent as well as in Sub-Saharan Africa, and have been highlighted in previous studies (26). Furthermore, women are reported to be engaged in more negative feelings/thoughts in comparison to child bearing mothers, and they also see their lives as meaningless. Continuous social comparison has a strong influence on the emotions of Pakistani women with primary infertility, and the subscale of Emotional Burden would be quite useful to assess this, which was found in previous studies (27). The social suffering of women with primary infertility due to negative social comparisons is in line with the findings of previous research in other countries, especially Asian and developing countries such as Bangladesh, Iran and India (28).

Another prominent factor of the Social Comparison Scale for Women with Infertility was Emotional Burden, as it directly measured the emotional experiences of the women. According to the psychometric properties of the scale, the women with higher scores on this factor had more emotional issues owing to their infertility. The present investigation is very indicative of the significant fact that negative emotions are practiced and experienced by the participants, ranging from crying to feelings of sadness, negative thoughts, low confidence, frustration, sorrow, embarrassment, self-pity, remorse, regret, anger, grief, tension, worry, doubt, shame feelings of rejection, boredom, solitude, depression, helplessness, hopelessness, powerlessness, melancholy, despair, distress, anguish, anxiety, irritability, envy, loss of pleasure/interest in daily activities and reduced sexual interest. Dissatisfaction with life has also been reported, with a strong desire for the child as a source of women’s pride. These items are similar to previous studies (25), which also highlighted the negative emotional consequences of infertility.

Subscale 3 of the SCS-WI was Personal Incapacity. A high score on this scale indicated the areas of personal lives of the women in which they were incapacitated due to infertility. For instance, feelings of physical and mental sickness, compact/reduced communication, feelings of inferiority, negligence in personal hygiene and care, fewer capabilities, feelings of physical weakness, fatigue, less resourcefulness, etc. constituted the main content of the items in this sub-scale. A high score on this subscale also shows that women are facing physical illness in the form of headaches, bodily pains, mental illness, and fatigue due to infertility. These findings are in line with previous research, which suggested that Pakistani women with infertility reported greater depression and somatic symptoms than women in the UK with infertility (5). Items of SCS-WI are based on experiences of Pakistani women with primary infertility and the theoretical framework of the social rank theory. Convergent validity of the SCS-WI was determined by correlating it with Social Comparison Scale (9) and was found to be good. Discriminant validity of SCS-WI was determined by correlating it with theoretically opposite scales such as the Satisfaction with Life Scale, and a significant negative correlation was found between both scales. The results coincide with the previous studies that have also shown a negative correlation between social comparison and satisfaction with life (28).

The Submissive Behavior Scale for Women with Infertility (SBS-WI) also appeared as a promising measure with internal consistency and a meaningful pattern of validity. Women with infertility experience all these emotions with more intensity, as previous research reported that women with infertility experience a higher degree of stress and fiery burden due to unfulfilled desire for a child (25). The items included in the scale are somehow similar to the issues reported in previous literature, including depression, acute stress, anxiety, interpersonal problems with friends and family, guilt, loss of self-identity, an increased sense of self-blame, and even in some cases, suicidal ideation (29). Pakistani women with primary infertility are subjected to systematic and progressive devaluation, as childbearing is a pre-requisite for women to strengthen their foothold in their husband’s home in the Pakistani culture, norms, philosophy and society (30).

SBS-WI (19) is a uni-dimensional scale, and some of the content of items of the scale appear to be similar to the Submissive Behavior Scale (16), and scale items are based on experiences of Pakistani women with primary infertility. Some of the items were eliminated after confirmatory factor analysis, such as “I avoid making eye contact with people due to infertility”, “I remain quiet on displeasing talk of people about me”, and “Due to infertility people take me for granted and assign their tasks to me”.

Convergent validity of SBS-WI (19) was determined by correlating with a similar construct i.e., the Submissive Behavior Scale (16), and was found to be good. Discriminant validity of SBS-WI was established by correlating it with a theoretically opposite scale such as the Self Assertiveness Scale, and a significant negative correlation was found (See Table 9). The results are in line with the study findings of previous research that showed a negative correlation between submissive behavior and assertive behavior, but at the same time, submissive behavior is not the mirror opposite of assertive behavior (16, 24) (The area of the relationship between assertive and submissive behavior needs to be explored further in the future).

**Table 9:** Convergent and discriminant validity of the SCS-WI and SBS-WI (*N* = 30).

Variables		1	2	3	4	5	6
1	SCS-WI	1	0.37*	−0.70**	0.61**	0.45*	−0.41*
2	SCS		1	−0.30*	0.32*	−0.35*	0.33*
3	SWLS			1	−0.71**	−0.56**	0.37**
4	SBS-WI				1	0.43*	−0.57**
5	SBS					1	−0.36*
6	SAS						1

SCS-WI, Social comparison Scale for Women with Infertility; SBS-WI, Submissive behavior Scale for Women with Infertility; SCS, Social Comparison Scale; SWLS, Satisfaction With Life Scale; SBS, Submissive Behavior Scale; SAS, Self Assertiveness Scale.

*p < 0.05, **p < 0.01.

The items of both scales reflect that the loss of one’s social rank is often escorted by the experience of one’s infertility. Thus, the opinion that most women’s ability to reproduce a child is linked to their status as women in every culture and society seems to be related to the findings of previous studies (27), which proposes that the social status of women with infertility can be significant in minimizing the effects of liberalization, with stigmatization and suffering associated with infertility. The study conducted (31) also suggests that negative social inferences about infertility are apparent in women of low status, especially in the emerging and developing world. In short, infertility impacts the overall quality of women’s life negatively.

The items of both scales reflect that loss of social rank is often accompanied by the experience of infertility. Thus, the observation that most women’s capability to reproduce is linked to their status as women in every culture and society seems to be related to the findings of previous studies (25).

### Implications

The newly developed scale seems to have promising reliability and sound convergent and discriminant validities. The scales developed in the study will help psychologists and counselors to assess the phenomenon of social rank demotion in which may contribute to psychopathologies, especially depression, in women with infertility in Pakistani women. The newly developed scales will further open new panoramas of exploration and examination in the domains of social comparison and submissive behavior, and their correlates.

### Limitations

Firstly, though the sample of the study was drawn from major cities of four provinces of Pakistan and represented women from various socioeconomic strata, women from rural areas and those who were not seeking treatment from any professionals were not included in this study. Secondly, the items of this newly constructed scale cannot be generalized to women with secondary infertility and men suffering from infertility.

## Conclusion

The Social Rank Scale for Women with Infertility with its two components (i.e., the Social Comparison Scale for Women with Infertility, SCS-WI, and the Submissive Behavior Scale for Women with Infertility, SBS-WI) are promising measures of social rank in Pakistani women with primary infertility, with sound psychometric properties, good items homogeneity, high reliability, and significant patterns of validity.

## Data availability statement

The raw data supporting the conclusions of this article will be made available by the authors, without undue reservation.

## Ethics statement

The studies involving human participants were reviewed and approved by the Ethical Review Committee of the Department of Psychology, GC University, Lahore Pakistan (Ref: REG-ACAD-ASRB-3/17). The patients/participants provided their written informed consent to participate in this study.

## Author contributions

AM: conception and design, data collection, data analysis, writing draft, and revision. SB: critical review, editing, and revision. SA: critical review, editing, and revision. All authors contributed to the article and approved the submitted version.

## Conflict of interest

The authors declare that the research was conducted in the absence of any commercial or financial relationships that could be construed as a potential conflict of interest.

## Publisher’s note

All claims expressed in this article are solely those of the authors and do not necessarily represent those of their affiliated organizations, or those of the publisher, the editors and the reviewers. Any product that may be evaluated in this article, or claim that may be made by its manufacturer, is not guaranteed or endorsed by the publisher.
