# Supplementary material for: Psychometric evaluation and validation of Urdu Social Rank Scale for women with infertility in Pakistan
Source: Front Psychiatry. 2023 Aug 30;14:1150941. doi: 10.3389/fpsyt.2023.1150941 (PMC10499518; doi:10.3389/fpsyt.2023.1150941)
Supplement: Supplementary file 1 [file Table_1.DOC]

**Table 1.** Standardized CFA Solution of Social Rank Scale (N = 210)

| *Model* | *IFI* | *TLI* | *CFI* | *df* | *RMSEA* | *p* | *x^2^* | *x^2^/df* |
| --- | --- | --- | --- | --- | --- | --- | --- | --- |
| Initial model | .84 | .83 | .84 | 898 | .08<.000 | 2039 | .52 | 2.27 |
| Final model | .09 | .09 | .09 | 881 | .06,.01 | 1576 | .13 | 1.78 |

*Note*. IFI = incremental fit index, CFI = comparative fit index, TLI = Tucker Lewis Index
