# Supplementary material for: Psychometric evaluation and validation of Urdu Social Rank Scale for women with infertility in Pakistan
Source: Front Psychiatry. 2023 Aug 30;14:1150941. doi: 10.3389/fpsyt.2023.1150941 (PMC10499518; doi:10.3389/fpsyt.2023.1150941)
Supplement: Supplementary file 2 [file Table_2.DOC]

**Table 2**. Overall Mean, Standard Deviation, Cronbach's Alpha, Maximum Score, Minimum

Score( N=210)

| Scale | K | Final Items retained | M(SD) | α | Min. | Max |
| --- | --- | --- | --- | --- | --- | --- |
| SCS-WI | 27 | 1-27 | 55.17(23.48) | .95 | 19 | 103 |
| Social distress | 11 | 5, 10, 13, 14, 15, 16, 17,  18, 19,25, 27 | 21.28(10.23) | .90 | 3 | 44 |
| Emotional burden | 7 | 2, 3, 21 , 22, 23, 24, 26 | 14.37(6.35) | .85 | 1 | 28 |
| Personal  Incapacities | 9 | 1, 4,6, 7, 8, 9, 11, 12, 20 | 17.43(7.99) | .87 | 2 | 33 |
| SBS-WI | 17 | 1-17 | 28.38(15.33) | .95 | 0 | 57 |
| SRS-WI | 44 | ------------------- | 81.48(36.70) | .84 | 19 | 153 |

*Note*. *k* = no. of items. *α = Cronbach’s alpha*
